# SPLEnic salvage and complications after splenic artery EmbolizatioN for blunt abdomINal trauma: the SPLEEN-IN study

**DOI:** 10.1186/s42155-020-00185-4

**Published:** 2020-12-07

**Authors:** Warren Clements, Tim Joseph, Jim Koukounaras, Gerard S. Goh, Heather K. Moriarty, Joseph Mathew, Tuan D. Phan

**Affiliations:** 1 Department of Radiology, Alfred Health, 55 Commercial Road, Melbourne, VIC 3004 Australia; 2 Department of Surgery, Monash University, Melbourne, Australia; 3 National Trauma Research Institute, Central Clinical School, Monash University, Melbourne, Australia; 4 Department of Trauma, Alfred Health, Melbourne, Victoria Australia

**Keywords:** Trauma, Spleen, SAE, Hemorrhage, Embolization

## Abstract

**Background:**

As an adjunct to non-operative management, splenic artery embolization (SAE) has been increasingly utilized throughout the world and is now the standard of care for hemodynamically stable patients. This study aimed to retrospectively assess the rate of splenic salvage and complications after SAE for blunt trauma at a level 1 trauma center using the 2018 update to the AAST criteria, and further sub-stratify the role of angiography in AAST grade III injuries with significant hemoperitoneum.

All patients between 1 January 2009 and 1 January 2019 who underwent blunt trauma and proceeded to embolization were included. Data was collected concerning initial injury grade, location of embolization, type of embolic material used, complications, and need for subsequent splenectomy. Technical success was defined as successful angiographic occlusion of the target artery at the conclusion of embolization. Clinical success was defined as splenic salvage at discharge. Vascular lesions were characterized including those with active bleeding, pseudoaneurysm, and arterio-venous fistula.

**Results:**

Two hundred thirty-two patients were included in the study. Treatments were performed at a median of 0 days (range 0–28 days) and the median AAST grade was IV (range III-V). Technical success was achieved in all patients. There were 13 complications (5.6%) consisting of re-bleed (9, 3.9%), infarction (3, 1.3%), and access site haematoma (1, 0.43%). Clinical success was achieved in 97% of patients with 7 patients requiring splenectomy after SAE (3.0%) at a median time of 4 days (range 0–17 days). Angiography in patients with grade III injuries identified 18 occult vascular injuries not identified at initial CT (*p* < 0.0001).

**Conclusions:**

The SPLEEN-IN study shows that treatment of intermediate-high grade blunt force traumatic splenic injuries using SAE resulted in a low rate of complication and splenic salvage in 97% of patients, providing a safe and effective treatment in stable patients. In addition, angiography of grade III injuries identified occult vascular lesions and may warrant treatment of select patients in this cohort.

**Level of evidence:**

Level 3.

## Introduction

Splenic artery embolization (SAE) has been increasingly utilized throughout the world (Roy et al. 2018). Splenic salvage for hemodynamically stable trauma patients is now standard of care (Patil et al. 2020). Splenic embolization is also relatively unique, in that it can offer both immediate hemodynamic control as well as preserving splenic function (Lukies et al. 2020; Aiolfi et al. 2017).

Modern trauma management requires a multi-disciplinary approach. Trauma physicians, surgeons, and interventional radiologists need to consider treatment in the context of the clinical scenario and intended goal. The first concept to consider is the spleen with vascular injury, including active bleeding or the presence of 1 or more pseudoaneurysms. In this circumstance, treatment is almost always indicated and this must be planned in addition to considering the overall severity of the injury (Quencer and Smith 2019). In focal vascular injuries, splenic embolization is often performed as distal to the hilum as possible to treat the vascular lesion but reduce the overall risk of infarction, however the choice of embolic location in this group is controversial particularly if there are multiple vascular lesions (Quencer and Smith 2019). A second scenario is high-grade splenic injuries without major vascular injury. Patients are often hemodynamically stable and treatment is targeted at reducing the overall rate of delayed re-bleed (Rong et al. 2017). In these circumstances, embolization proximal to the hilum is usually performed. In some cases where there is a vascular lesion and a high-grade injury, a tandem embolization (both proximal and distal) may be employed.

The severity of injury may be graded according to a variety of different injury classification systems. Arguably the most widely used is the American Association for the Surgery of Trauma (AAST) which first released a grading system in 1994 (Moore et al. 1989). A 2018 manuscript which included authors from the patient assessment committee of the AAST, put forward a revised grading system whereby it was suggested that the presence of vascular injury be incorporated into the original AAST system to more accurately reflect the need for NOM adjuncts such as SAE in these patients (Kozar et al. 2018).

One of the biggest problems facing practitioners is the heterogeneity in the endovascular treatment offered to these patients. Who to treat, when to treat, which embolic to use, and where to employ the chosen embolic harbors considerable debate and controversy, particularly for AAST grade III injuries.

At The Alfred Hospital, a level 1 trauma center in Melbourne, Australia, we consider angiography for patients with high grade injury (AAST IV and V). In addition, we consider angiography for patients with intermediate-grade injuries (AAST III) where there is significant hemoperitoneum as defined by the presence of blood in three or more abdominal quadrants on CT, although we acknowledge that this is an area of controversy.

This study aimed to retrospectively assess the rate of splenic salvage after blunt trauma using our protocol. In addition, we aimed to sub-stratify the angiographic findings in patients with AAST III injuries to identify whether our protocol can clarify the role of treatment in this controversial cohort of trauma patients.

## Material and methods

### Ethics

Approval was provided by The Alfred Hospital Human Research and Ethics Committee, number 361/19. For this retrospective analysis, individual patient consent was not required.

### Patient identification

The study covered a 10-year period from 1 January 2009 to 1 January 2019. Patients were identified through the Radiology Information System (RIS). Information including demographics, treatment, and complications were obtained from a combination of RIS, Picture and Communications Archive (PACS), and the Electronic Medical Record (EMR). Splenic injuries were graded on CT by 2 radiologists and any discrepancies were mediated by an independent interventional radiologist before being included.

### Inclusion criteria and endpoints

All patients over the age of 16 who underwent SAE after blunt trauma were included. Patients were excluded if the injury was penetrating, if an angiogram was performed but no embolization was employed, or if SAE was performed for non-traumatic reasons. Complications were defined according to the CIRSE classification system (Filippiados et al. 2017). Potential complications included abscess, infarction/post-embolization syndrome, access site complication, access vessel dissection, and splenectomy.

### Data collection

Data collected included age, gender, injury severity score (ISS), time from injury to embolization, AAST trauma grade (2018 classification), vascular injury at CT, vascular injury at angiogram, location of embolization, type of embolic material used, complication, time for complication to occur, need for splenectomy after SAE, and time for splenectomy to occur after SAE. Vascular injury was defined as the presence of active bleed, pseudoaneurysm, or arteriovenous fistula. Data was collected during the index admission and up to 30 days after the traumatic event.

### Embolization definition

**Fig. 1:**
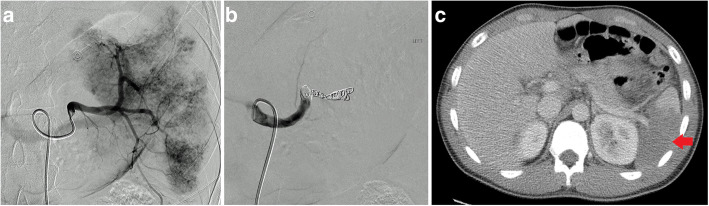
Catheter angiography from the splenic artery showing high grade parenchymal injury **a** successfully treated with proximal embolization **b** and patient with infarct after proximal embolization (arrow) **c**

**Fig. 2:**
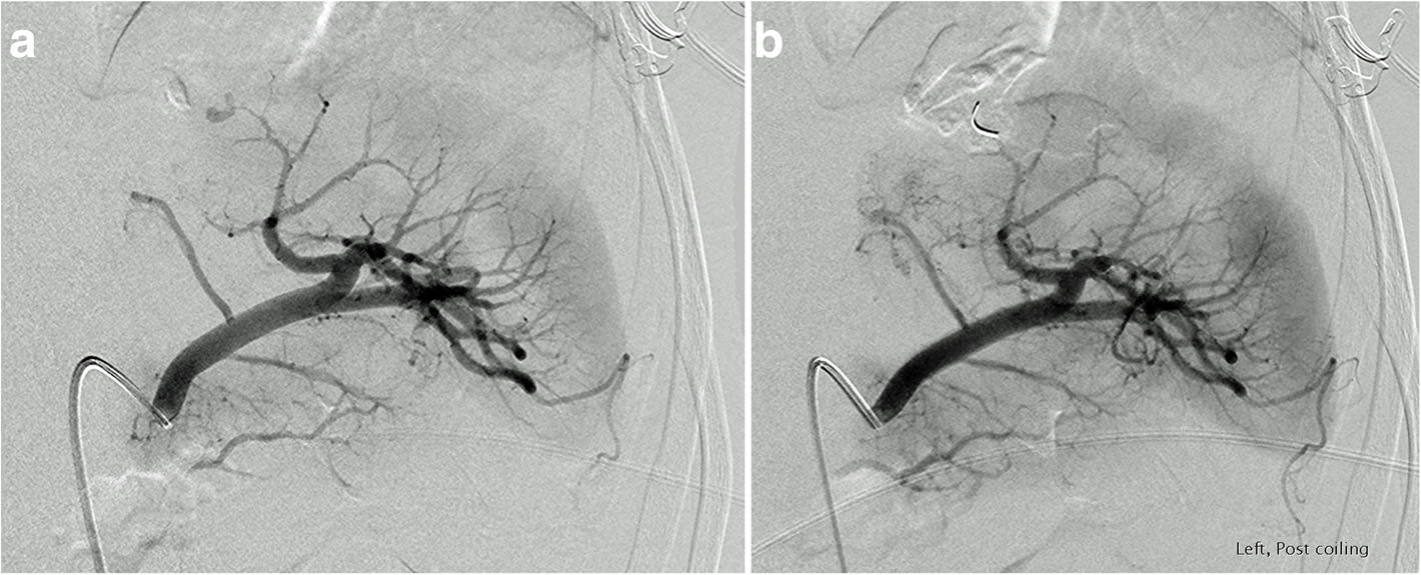
Selective catheter angiography from an upper pole splenic artery showing focal parenchymal injury **a**, successfully treated with distal embolization **b**

For the purposes of this manuscript, “proximal embolization” was defined as large vessel occlusion proximal to the splenic hilum but distal to the dorsal pancreatic artery (Fig. 1a and b). “Distal embolization” was considered embolization of selected splenic artery branches distal to the hilum (Fig. 2a and b). “Tandem embolization” refers to a procedure where distal embolization was performed for focal vascular lesion followed by concurrent proximal embolization.

### Outcome definition

Technical success was defined as angiographic occlusion of the target artery at the conclusion of the treatment. Clinical success was defined as splenic salvage after SAE.

### Embolization technique

All procedures were performed by one of 8 fellowship-trained interventional radiologists, or an advanced trainee under supervision. According to operator preference, embolization is performed using pushable coils (0.018“ or 0.035” Cook Nester or Cook Tornado, Cook Medical, Bloomington, USA), amplatzer vascular plug (St Jude Medical, Plymouth, USA), microvascular plug (Medtronic, Dublin, Ireland), EOS (Artventive, San Marcos, USA), or gelatin sponge.

### Statistical analysis

Data was de-identified and analyzed using Microsoft Excel (Microsoft, USA) with the Real Statistics Resource Pack software (Release 6.8) (Zaiontz 2020). Data was summarized using mean and standard deviation, median and range, or frequency and percentage as appropriate to the type of data. Using the Mann-Whitney U test, a two-sided *p*-value less than 0.05 was chosen to indicate statistical significance.

## Results

### Patient demographics

During the defined study time, 232 patients met inclusion criteria for the study. The mean age was 40 years and 80.1% were male. Treatments were performed at a median time of 0 days (day of injury) and the median AAST injury grade was IV. On initial trauma CT, 59% of patients had a vascular injury while at angiography 79% showed a vascular injury (*p* = 0.001). Table 1 summarizes patient and treatment demographics.

**Table 1:** Patient and procedure demographics

Number of embolizations	232
Age (mean, SD)	40 (18.7)
Male gender (number, percentage)	185 (80.1%)
Time to embolization after injury in days (median, range)	0 (0–28)
AAST^a^ injury grade (median, range)	4 (3–5)
Evidence of vascular injury at CT (number, percentage)	137 (59.0%)
Evidence of vascular injury at embolization (number, percentage)	184 (79.3%)
Proximal embolization (number, percentage)	176 (75.9%)
Use of pushable coils (number, percentage)	197 (84.9%)
Injury severity score (median, range)	22 (4–66)
Complications (number, percentage)	13 (5.6%)
Time to complication in days (median, range)	2.1 (0–7)
Splenectomy after embolization (number, percentage)	7 (3.0%)
Time to splenectomy (median, range)	4 (0–17)

^a^*AAST* American Association for the Surgery of Trauma

**Fig. 3:**
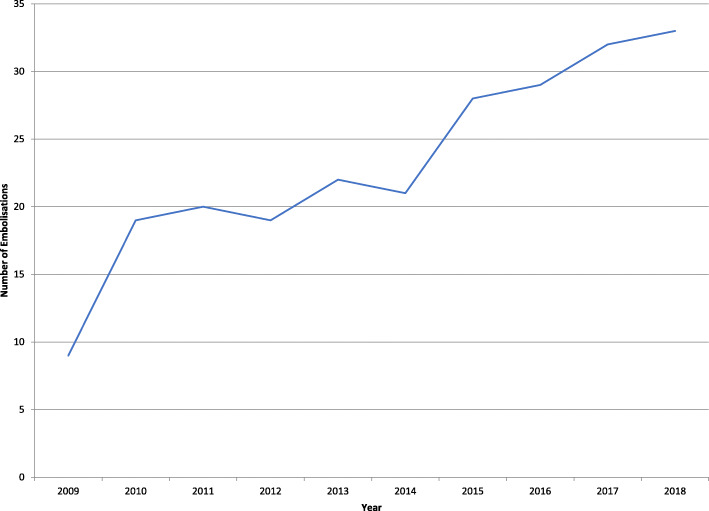
Ten-year trend of increasing number of SAE procedures at our institution for blunt abdominal trauma

Only 9 embolizations were performed in 2009 while 33 were performed in 2018 with the increasing trend between these years shown in Fig. 3.

### Success and complications

Technical success was achieved in 100% of patients. There were 13 complications in total (5.6%) including 1 CIRSE grade 1, 3 CIRSE grade 2, 3 CIRSE grade 3, and 6 CIRSE grade 4. There were 7 patients (3.0%) who underwent splenectomy after embolization meaning the splenic salvage rate was 97%. Table 2 outlines the individual demographics of patients who either experienced a complication and/or underwent subsequent splenectomy. Compared with the overall cohort, there was no significant difference in the group who experienced a complication in terms of ISS (*p* = 0.52), age (*p* = 0.41), gender (*p* = 0.27), or vascular injury at CT (*p* = 0.29).

**Table 2:** Patients who experienced complications and/or underwent splenectomy after SAE

Patient number	Age	Gender	ISS	AAST injury grade	Vascular lesion on CT?	Vascular lesion on angiogram?	Location of embolization	Type of embolic used	CIRSE complication status (Filippiados et al. 2017)	Complication type	Splenectomy required	Days to splenectomy after trauma
1	17	Male	16	4	No	Yes	Proximal	Pushable coils	2	Infarction	No	N/A
2	22	Female	33	4	Yes	Yes	Proximal	Plug (AVP IV)	2	Infarction	No	N/A
3	48	Male	17	4	Yes	No	Proximal	Pushable coils	3	Re-bleed	No	N/A
4	30	Male	38	5	Yes	Yes	Proximal	Plug (AVP IV)	**N/A**^a^	**N/A**^a^	Yes	0
5	55	Male	29	5	Yes	Yes	Proximal	Plug (AVP IV)	3	Re-bleed	No	N/A
6	54	Female	41	5	Yes	Yes	Tandem	Pushable coils and gelfoam	3	Re-bleed	No	N/A
7	29	Male	21	5	Yes	Yes	Proximal	Pushable coils	4	Re-bleed	Yes	0
8	89	Male	10	4	Yes	Yes	Proximal	Pushable coils	1	Groin hematoma	No	N/A
9	48	Female	34	5	No	Yes	Proximal	Pushable coils	4	Re-bleed	Yes	4
10	31	Male	34	5	No	Yes	Proximal	Pushable coils	4	Re-bleed	Yes	5
11	37	Female	57	4	No	Yes	Proximal	Plug (Eos)	4	Re-bleed	Yes	10
12	78	Male	20	4	No	No	Proximal	Pushable coils	4	Re-bleed	Yes	17
13	53	Male	20	5	Yes	Yes	Proximal	Pushable coils	4	Re-bleed	Yes	3
14	38	Female	16	5	Yes	Yes	Proximal	Pushable coils	2	Infarction	No	N/A

*ISS* injury severity score

*AAST* American Association for the Surgery of Trauma

^a^patient 4 underwent planned splenectomy after pelvic embolization and the spleen was embolized pre-operatively. This was not considered a complication of embolization

### Choice of embolic

The majority of procedures (84.9%) employed pushable fibered coils as shown in Table 3. A vascular plug was used in 6%. Cases using combination embolic (5.6%), detachable coils (2.2%), and gelatin sponge (1.3%) were less frequent. All combination embolic cases consisted of pushable coils and gelatin sponge. The overall rate of complications was not significantly different between the groups (*p* = 0.079).

**Table 3:** Use of different embolic agents, and association with complication and need for splenectomy

Type of embolic	Number	Percentage	Complications	Splenectomy
Pushable coils	197	84.9	9*	5*
Detachable coils	5	2.2	0*	0*
Vascular plug	14	6.0	4*	2*
Gelatin sponge	3	1.3	0*	0*
Combination of embolic	13	5.6	1*	0*

*p > 0.05

**p < 0.05

### Location of embolization

Embolizations were proximal to the hilum in 176 patients (75.9%) with a complication rate of 6.8% in this cohort, and 7 patients (4.0%) required subsequent splenectomy. There were no complications in the 35 patients (15.1%) who underwent distal embolization. Tandem embolization was used in 21 patients (9.0%) and only 1 (0.43%) experienced a complication but did not require splenectomy. No significant difference in the rate of complications (*p* = 0.69) or splenectomy (*p* = 0.32) was identified based on the embolization location.

### AAST grading

Using the updated 2018 AAST grading system, Table 4 outlines the number of treatments and patient demographics based on each injury grade. There were 41 patients with AAST grade III injury, 109 patients with grade IV injury, and 82 patients with grade V injury. Patients with grade IV injuries had a significantly lower mean age than for the other grades, *p* = 0.01. There was a higher proportion of males in the group of patients with grade III injuries than for other groups, *p* = 0.027. There was a higher rate of vascular injuries in the grade V patient group (*p* < 0.0001). No significant difference was seen between the groups comparing ISS (*p* = 0.21), proportion of proximal embolizations (*p* = 0.55), time to embolization (*p* = 0.81), complication rate (*p* = 0.085), or splenectomy rate (*p* = 0.11).

**Table 4:** Comparison of AAST subgroup demographics (2018 revision) and outcomes

hba	Age (mean, SD)	ISS (median, range)	Male gender (number, percentage)	Vascular injury at CT (number, percentage)	Number of embolizations	Proximal embolization (number, percentage)	Time to embolization (median, range)	Complications (number, percentage)	Splenectomy (number, percentage)
Grade III	44 (19.4)*	22 (9–59)*	38 (93%)**	N/A^a^	41	33 (87%)*	0 (0–18)*	0 (0%)*	0 (0%)*
Grade IV	36 (17.0)**	22 (4–57)*	80 (73%)*	48 (44%)*	109	85 (78%)*	0 (0–21)*	6 (5.5%)*	2 (1.8%)*
Grade V	43 (19.7)*	22 (5–66)*	67 (82%)*	74 (90%)**	82	58 (71%)*	0 (0–28)*	7 (8.5%)*	5 (8.5%)*

*ISS* injury severity score

*AAST* American Association for the Surgery of Trauma

*p > 0.05

**p < 0.05

^a^vascular injury is not possible with grade III classification

### AAST grade III

Of the 41 patients who were graded as AAST III based on their initial CT but proceeded to angiography/SAE, 18 of these patients showed evidence of a vascular injury at angiography compared to 0 who showed evidence of vascular injury at CT (*p* < 0.0001). There were no complications and no patients who required splenectomy in this cohort.

## Discussion

The number of splenic embolizations has been steadily increasing in frequency at our institution, and similarly reflected in data from other institutions including from different countries (Roy et al. 2018). There are many factors that may contribute to this. From the perspective of our network, our hospital is the largest Level 1 center in the Victorian State Trauma system which has preprogrammed responses built in to patient referral and treatment processes and is supported by efficient ground and air ambulance including a 24-h helipad. This has decreased mortality and also time to definitive hemorrhage control (Cameron et al. 2008). On arrival to the trauma center, a multidisciplinary trauma team approach to damage control resuscitation has decreased the time to stabilization, access to CT, and definitive hemorrhage control. This may allow for endovascular rather than emergency operative intervention (Matsumoto et al. 2015). In addition, a dedicated interventional radiology on-call roster allows for rapid activation of services. These factors support a median time to treatment of 0 days. The increasing trend also reflects the increasing abundance of supportive data towards the role of SAE in supporting NOM in blunt trauma including recent systematic reviews (Moore et al. 1989; Schnüriger et al. 2011).

The demographic in this study with a mean age of 40 and predominantly males, is in keeping with the expected demographic of road traffic trauma (Ferrah et al. 2019). The median injury severity score of 22 also reflects the complexity of high-energy multi-system trauma at our institution (Ferrah et al. 2019). The demographics in both the overall cohort and for those who experienced a complication were similarly matched.

The overall rate of complications in this study was low at 5.6% and occurred at a median time of 2.1 days after the treatment. In addition, only 3% of patients proceeded to splenectomy at a median time of 4 days which is low, and in keeping with the rate shown in prior literature (Moore et al. 1989; Schnüriger et al. 2011; Davies and Wells 2019; Hughes et al. 2017). Of the 7 patients requiring splenectomy after embolization, 1 patient was a 30-year-old male who presented after a motor vehicle accident with multiple injuries (ISS 38) including grade V splenic injury, hollow viscus injury, and pelvic bleeding on CT scan. The patient was emergently transferred to the angiography suite before the operating theatre, where pelvic embolization was performed, and a decision was made between the interventional radiologist and trauma surgeon to embolize the spleen proximally to facilitate safe transfer to the operating suite where splenectomy was performed as a pre-planned procedure. While this has been included in the analysis as a splenectomy after embolization, in the opinion of the authors it does not reflect a complication of the embolization itself.

With the update to the AAST classification in 2018 to incorporate splenic artery vascular injury, 82% are now considered high-grade (AAST IV or V). This compares to 126 high-grade injuries (54%) if our patients were graded per the previous 1994 AAST classification. The grade V cohort showed a significantly higher rate of vascular injury than for the lower grade groups which is expected given the severity of injury to meet the grade V criteria. The rate of splenectomy in the grade V cohort of 8.5% is also low and comparable to literature including the systematic review of Rong et al. which included 10 studies and 876 patients (Moore et al. 1989). It is encouraging to see that of all patients with grade IV splenic injury or lower, only 2 of 150 patients (1.3%) required splenectomy after SAE.

Our practice to strongly consider angiography in patients who have AAST grade III injury and three or more quadrant hemoperitoneum (41 patients, 17.6%) is acknowledged to be a controversial decision. However, as shown in this study there was a significant increase in the number of vascular injuries identified at angiography compared to those identified at CT in patients who were deemed to have AAST III injury based on their trauma CT scan. It is for this reason that considering angiography in the grade III cohort is likely to identify patients who are at increased risk of re-bleed. The absolute risk however is difficult to quantify without a prospective and randomized design, which may not be feasible. The effect of these results is to further sub stratify this patient group beyond the AAST classification where it is possible that occult vascular injuries are the reason for those who do subsequently re-bleed with a grade III injury. In addition, it can be argued that the low rate of complications in the AAST III cohort of 0% and preservation of splenic function (Lukies et al. 2020; Schimmer et al. 2016) further warrants such consideration for treatment. The presence of vascular injury in the grade III cohort from this study also supports the recent changes to AAST to acknowledge the importance of vascular injury in their classification system. In addition, with a median ISS of 22 and as high as 59 in the grade III cohort, a diminished risk of re-bleed after embolization is often appreciated in complex multi-trauma patients where the cause of abnormal or changing physiology can be hard to pinpoint.

In the whole cohort, the choice of proximal embolic location (75.9%) and pushable fibred coils (84.9%) reflects a decision at our institution to provide a cost-effective solution for our patients in an institution that is government-funded (Yip et al. n.d.). In many cases, proximal embolization also offers a rapid option to provide treatment even with significant anatomic tortuosity, anatomic variation, vasospasm, and/or underlying vascular disease which may inhibit or prolong attempts at distal embolization. Such choices are at the discretion of the treating interventional radiologist at the time of embolization even though a range of catheters and embolic materials are always in stock.

It is encouraging to see that there is no significant difference in the rate of complication or splenectomy based on the choice of embolic material or the embolic location in this study. The analysis of Rong et al. suggested a higher rate of complications with the use of gelatin sponge and this has also been shown in other studies (Moore et al. 1989; Abada and Golzarian 2007). However, as the number of cases using gelatin sponge in our study was extremely low, it is not possible to correlate with their findings reliably. While it is unusual to see zero complications in the distal embolization cohort, the small number of patients in this cohort (35, 15.1%) which was heavily biased towards proximal embolization may account for this.

The authors acknowledge a number of limitations with this study. This is a retrospective audit and as such lacks patient-level clinical data in which the trauma setting it is arguable most vital. As such, we have used AAST and ISS to risk-stratify patients. As a single-center study, our practice reflects the internal structure of our trauma and interventional radiology teams and is also impacted by the nearby position of emergency/trauma and imaging services. This may not necessarily reflect how other hospitals are structurally designed. We also acknowledge that AAST grade was determined at initial CT and while treatment was performed based on a combination of CT and angiographic findings, the grade was not changed after angiography for the purposes of this analysis. We acknowledge the heterogeneity in the way embolizations were performed in our cohort and that there has been grouping of individual patients regardless of the fine detail of splenic parenchymal injury variation. The choice of embolic and choice of embolic location is also heavily skewed to proximal embolization with pushable coils, leaving smaller numbers for the other groups. This will reduce the statistical reliability of analysis. In addition, all embolics are different even for pushable coils and the effectiveness between 0.018″, 0.035″, and different types or lengths of coils in each size was not measured. Nevertheless, our results indicate that proximal splenic embolization is a robust strategy that can be successful despite technical differences and this is supported in previous systematic reviews which also suffer from similar heterogeneity in data (Moore et al. 1989; Schnüriger et al. 2011).

In conclusion, the SPLEEN-IN study shows that treatment of intermediate-high grade blunt force traumatic splenic injuries using SAE results in a low overall rate of complication and splenic salvage in 97% of patients, providing a safe and effective adjunct to NOM in these patients. Splenic salvage after SAE increases to 98.7% of patients with a grade IV injury or lower. These results can be achieved with lower cost pushable coils and proximal splenic embolization in most patients.

The SPLEEN-IN study also shows that embolization of grade III injuries in certain patients is safe, and that proceeding to angiography in this cohort can identify vascular injuries initially occult on CT. The results support the recent changes to the AAST classification where the importance of splenic vascular injury is now acknowledged.

## Data Availability

The datasets generated and/or analyzed during the current study are not publicly available as this was not a part of the IRB approval process, please contact the corresponding author for additional information.

## Abbreviations

NOM: Non-operative management
SAE: Splenic artery embolization
CT: Computed tomography
AAST: American Association for the Surgery of Trauma
ISS: Injury severity score
RIS: Radiology information system
PACS: Picture and communication archive
EMR: Electronic medical record

## References

[CR1] Abada HT, Golzarian J (2007). Gelatine sponge particles: handling characteristics for endovascular use. Tech Vasc Interv Radiol.

[CR2] Aiolfi A, Inaba K, Strumwarrer A (2017). Splenic artery embolization versus splenectomy. Analysis for early in-hospital infectious complications and outcomes. J Trauma Acute Care Surg.

[CR3] Cameron PA, Gabbe BJ, Cooper DJ (2008). A statewide system of trauma care in Victoria: effect on patient survival. Med J Aust.

[CR4] Davies J, Wells D (2019). Splenic artery embolisation in trauma: a five-year single-Centre experience at a UK major trauma Centre. Trauma..

[CR5] Ferrah N, Cameron P, Gabbe B (2019). Trends in the nature and Management of Serious Abdominal Trauma. World J Surg.

[CR6] Filippiados DK, Binkert C, Pellerin O (2017). Cirse quality assurance document and standards for classification of complications: the Cirse classification system. Cardiovasc Intervent Radiol.

[CR7] Hughes J, Scrimshire A, Steinberg L (2017). Interventional radiology service provision and practice for the management of traumatic splenic injury across the regional trauma networks of England. Injury..

[CR8] Kozar RA, Crandall M, Shanmuganathan K (2018). Organ injury scaling 2018 update: spleen, liver, and kidney. J Trauma Acute Care Surg.

[CR9] Lukies MW, Kavnoudias H, Zia A, Lee R, Bosco JJ, Joseph T, Clements W (2020) Long term immune function following splenic artery embolisation for blunt abdominal trauma. Cardiovasc Intervent Radiol. 10.1007/s00270-020-02627-x 10.1007/s00270-020-02627-x 32875434

[CR10] Matsumoto J, Lohman BD, Morimoto K (2015). Damage control interventional radiology (DCIR) in prompt and rapid endovascular strategies in trauma occasions (PRESTO): A new paradigm. Diagn Interv Imaging.

[CR11] Moore EE, Shackford SR, Pachter HL (1989). Organ injury scaling: spleen, liver, and kidney. J Trauma.

[CR12] Patil MS, Goodin SZ, Findeiss LK (2020). Update: splenic artery embolization in blunt abdominal trauma. Semin Interv Radiol.

[CR13] Quencer KB, Smith TA (2019). Review of proximal splenic artery embolization in blunt abdominal trauma. CVIR Endovasc.

[CR14] Rong J-J, Liu D, Liang M (2017). The impacts of different embolization techniques on splenic artery embolization for blunt splenic injury: a systematic review and meta-analysis. Military Med Res.

[CR15] Roy P, Mukherjee R, Parik M (2018). Splenic trauma in the twenty-first century: changing trends in management. Ann R Coll Surg Engl.

[CR16] Schimmer JAG, van der Steeg AF, Zuidema WP (2016). Splenic function after angioembolization for splenic trauma in children and adults: a systematic review. Injury.

[CR17] Schnüriger B, Inaba K, Konstantinidis A, Lustenberger T, Chan LS, Demetriades D (2011). Outcomes of proximal versus distal splenic artery embolization after trauma: a systematic review and meta-analysis. J Trauma.

[CR18] Yip H, Skelley A, Morphett L, Mathew J, Clements W The cost to perform splenic artery embolisation following blunt trauma: analysis from a level 1 Australian trauma Centre. Injury. 10.1016/j.injury.2020.09.039 10.1016/j.injury.2020.09.039 32962832

[CR19] Zaiontz C (2020). Real Statistics Using Excel.

